# Comprehensive Literature Review: Recent Advances in Diagnosing and Managing Patients with Poorly Differentiated Thyroid Carcinoma

**DOI:** 10.1155/2013/317487

**Published:** 2013-02-12

**Authors:** Jack Hannallah, Jessica Rose, Marlon A. Guerrero

**Affiliations:** ^1^Department of Surgery, University of Arizona, Tucson, AZ, USA; ^2^The University of Arizona Cancer Center, Tucson, AZ, USA; ^3^Division of Surgical Oncology, Department of Surgery, University of Arizona, P.O. Box 245131, Tucson, AZ 85724-5131, USA

## Abstract

Poorly differentiated thyroid carcinomas are a rare form of thyroid carcinomas; they display an intermediate behavior between well-differentiated and anaplastic thyroid carcinomas. PDTCs are more aggressive than the well-differentiated, but less aggressive than the undifferentiated or anaplastic, forms. No clinical features can accurately diagnose poorly differentiated thyroid carcinomas. Thus, the results of histocytology, immunohistochemistry, and molecular genetics tests aid in diagnosis. Given the aggressiveness of poorly differentiated thyroid carcinomas and the poor survival rates in patients who undergo surgery alone, a multimodality treatment approach is required. We conducted a comprehensive review of the current diagnostic and therapeutic tools in the management of patients with poorly differentiated thyroid carcinomas.

## 1. Introduction

Thyroid carcinomas are categorized along a continuum, usually based on the degree to which neoplastic parenchymal cells mimic the corresponding normal parenchymal cells, both in cellular morphology and functionality [1]. At one extreme, well-differentiated thyroid carcinomas (WDTCs), like the papillary and follicular thyroid forms (PTCs and FTCs), typically confer a favorable prognosis. However, at the opposite end of the spectrum, undifferentiated carcinomas, like anaplastic thyroid carcinomas (ATCs), are aggressive and rapidly fatal. Poorly differentiated thyroid carcinomas (PDTCs) exhibit a unique histologic architecture and morphologic changes that result in tumor behavior more aggressive than that of WDTCs but less aggressive than that of ATCs [2–4]. Although the literature collectively concurs on the clinical significance and existence of PDTCs the morphological cellular features for diagnosing PDTCs are still a topic of controversy.

The term “PDTC” was introduced and defined in the 1980s by Sakamoto et al. [5] and Carcangiu et al. [6]. Those two groups described similar neoplastic tumors, but with varying diagnostic criteria. This issue still holds true: some authors define PDTCs on the basis of unique histologic architectural growth patterns (insular, trabecular, or solid); other authors, on the basis of aggressive histologic behaviors (necrosis, increased mitotic rates, and vascular invasion). Recently, several studies have statistically validated that diagnosing PDTCs on the basis of biological behaviors (rather than on growth patterns) demonstrates greater clinical and prognostic significance [4, 7].

In light of the increased clinical significance of PDTCs and the lack of unifying diagnostic criteria, a consortium of experts met in Turin, Italy, in 2006, and proposed a homogenous set of diagnostic criteria for PDTCs [3]. Largely, they retrospectively established criteria based on both histologic architectural grade and cytomorphologic features of neoplastic thyroid cells. Establishing the criteria helped clarify and reinforce the principles that PDTCs are a unique and separate pathologic process and that such patients require clinical care that is different from that for WDTC patients. 

We conducted a comprehensive review of the current diagnostic and therapeutic tools in the management of poorly differentiated thyroid carcinomas.

## 2. Histocytology

Technically, PDTCs originate from either follicular or papillary epithelial cells and typically reveal a trabecular, insular, and/or solid (TIS) histomorphologic pattern [3]. PDTCs are classically characterized by the insular growth pattern, first described by Carcangiu et al., displaying large cellular nests with small round nuclei typically surrounded by a fibrovascular network [2, 4, 8–11]. These can be categorized as two major subtypes, the insular and insular-like carcinomas. Moreover, PDTCs can present as either a mixture of the three patterns or as one independent pattern. However, purely insular carcinomas are infrequent; insular-like carcinomas featuring trabecular or solid architecture are much more common [8, 12]. Most authors would agree that hematoxylin and eosin stain results solely demonstrating the aforementioned histomorphologic patterns are not enough to establish a diagnosis of PDTC; more morphologic features must be identified. 

Many investigators have attempted to define PDTCs by cytology tests because of their increased clinical prognostic value. PDTCs are often cellular, with scant colloid, and lack both nuclear pleomorphism and high-grade atypia [8, 9]. Furthermore, mitoses, high nuclear/cytoplasmic (N/C) ratio, loss of cellular polarity, and hyperchromasia are common cytologic features of PDTCs [1, 8, 9]. In a statistical analysis of 32 cytomorphologic features in 40 histologically proven cases of PDTC, Bongiovanni et al. demonstrated that the following 4 cellular features from a fine-needle aspirate (FNA) were predictive of PDTC: an TIS growth pattern; a high N/C ratio; a single-cell pattern; and severe cellular crowding [8]. Moreover, in a clinicopathologic study of 58 patients, Hiltzik et al. demonstrated that necrotic and mitotic features are better able to stratify patients into different prognostic categories than growth patterns alone [7].

**Figure 1:**
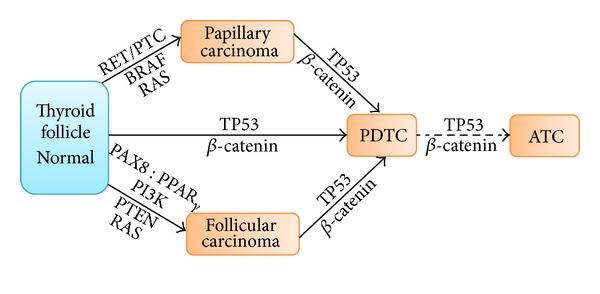
Poorly differentiated thyroid carcinomas (PDTCs) can develop de novo or from papillary or follicular carcinomas, through genetic alterations, possibly progressing into anaplastic thyroid carcinomas (ATCs).

PDTCs can develop from WDTCs or de novo entities (Figure 1). Therefore, it is difficult to determine how much of an TIS pattern is required on an FNA sample to make the diagnosis of PDTC [3, 13]. Bongiovanni and Faquin found that WDTCs with a 10% or greater TIS component confer a more aggressive clinical course and a poorer prognosis than WDTCs without PDTC features [9]. Several studies have proposed parameters of architectural patterns of >10%, >40%, and >70% to establish the diagnosis of PDTC; however, a standard minimum cutoff point has yet to be determined or established [10, 14]. It is worth mentioning that Volante et al., in a study of 183 patients with TIS patterns, did not find a statistically significant difference in the overall survival rate of patients with only a minor TIS component (of 10% to 50%) versus patients with a well-represented TIS component (of 50% to 75%) or a virtually “pure” TIS component (>75%) [4]. Although survival rates were statistically insignificant, patients with a minor TIS architectural component had a trend towards a more favorable prognosis [4]. Moreover, in a 40-patient case series, Pulcrano et al. did not find a correlation between a particular PDTC architectural subtype and metastasis-free survival [15].

**Figure 2:**
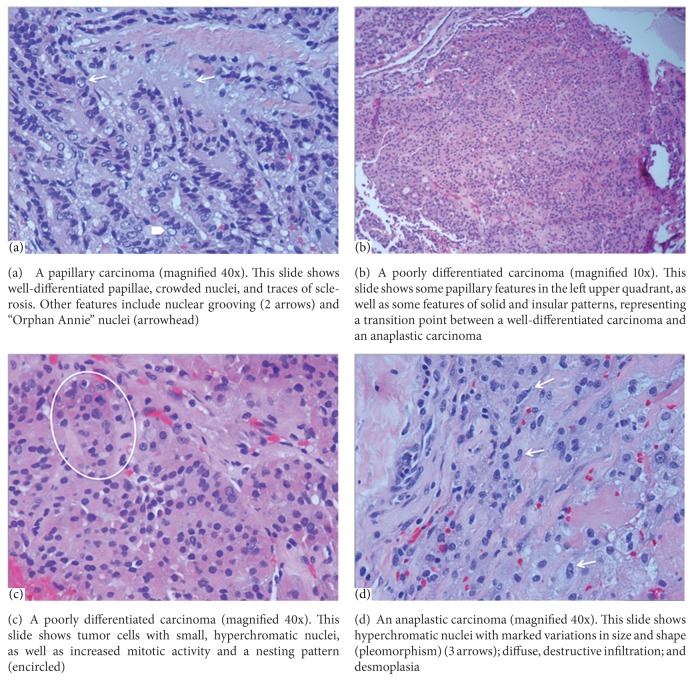
These slides illustrate the various histologic features of well-differentiated thyroid carcinomas (WDTCs), poorly differentiated thyroid carcinomas (PDTCs), and anaplastic thyroid carcinomas (ATCs).

Although defining PDTCs on cytomorphologic features is less expensive, internationally feasible, and relatively reliable, immunohistochemical stains and molecular genetics are now playing an increased role in diagnosing PDTCs. So, given the limited resources of various medical institutions around the world, the 2006 Turin proposal remains, arguably, the most accepted unifying and encompassing criteria for diagnosing PDTCs. According to that proposal, PDTCs are defined by: (1) the presence of an TIS architecture (2) with at least one of the following features present: convoluted nuclei; mitotic activity greater than 3 per 10 high-power fields; or tumor coagulative necrosis; and (3) absence of the conventional nuclear features of papillary thyroid carcinoma [3] (Figure 2).

## 3. Immunohistochemistry

Immunohistochemical stains have been widely considered by many authors to increase the diagnostic predictability of PDTCs. No specific molecular marker or immunohistochemical stain is specific to the detection of PDTCs, yet test results can rule out other thyroid carcinomas [8, 13]. For instance, PDTCs are negatively immunoreactive to calcitonin, carcinoembryonic antigen (CEA), chromogranin, and synaptophysin; such results reliably rule out medullary thyroid carcinomas and neuroendocrine tumors [8, 9]. Similarly, PDTCs are not immunoreactive to hematopoietic cellular markers, such as B-lymphocyte antigen (CD19 and CD20) and plasma cell marker (CD138); such results help exclude lymphoproliferative disorder [8].

In a small case series, Bongiovanni et al. demonstrated that PDTC samples were strongly immunohistochemically reactive to thyroglobulin (Tg), thyroid transcript factor 1 (TTF-1), and a cytokeratin cocktail [13, 14]. To further differentiate PDTCs from other thyroid carcinomas, Patel et al. showed that ATCs are not immunoreactive to thyroglobulin [14]. PDTCs may also be weakly immunoreactive (<50% of cases) to HBME-1, galectin-3, CD44v6, and Bcl-2. However, such stains alone cannot definitively differentiate PDTCs from WDTCs [13, 16]. But the unique genetic behavior of the cadherin/catenin complex has improved the ability to differentiate PDTCs from WDTCs. 

*Cadherins* aid in cell-to-cell adhesion; loss of adhesion correlates with an increased loss of differentiation in most carcinomas derived from epithelial cells [17]. Rocha et al. observed that WDTCs retained E-cadherin/catenin expression, whereas PDTCs displayed heterogeneous loss of E-cadherin and retained catenins [17]. Additionally, Rocha et al. concluded that the progressive loss of E-cadherins (rather than the previously assumed genetic mutation in catenins) is the more “crucial event” in determining the level of differentiation in thyroid carcinomas [17]. Nevertheless, according to many other studies, *β*-catenin mutations are significantly more specific for PDTCs—present, on average, in 25% of PDTC cases (range, 20% to 32%) [16, 18, 19].

*Ki67 antigen* is another immunohistochemical marker helpful in diagnosing PDTCs [15, 17]. A nuclear protein, Ki67, has an important role in cellular proliferation through ribosomal RNA transcription. Since PDTCs have a high mitotic activity, Ki67 antigen labeling can help to not only diagnose PDTCs but also to enumerate the extent of mitotic involvement [15, 20]. 

The *IMP3 protein* may also have value in diagnosing PDTCs as well as serving as a prognostic indicator [21]. Asioli et al. determined that IMP3 expression in PDTC patients is a negative prognostic indicator associated with an increased risk of death, lymph node metastasis, and distant metastasis [21]. Moreover, Asioli et al. stated that PDTCs are often associated with HBME-1, Ki67, and p53, but those markers are limited in their ability to specifically diagnose PDTCs [21]. To date, IMP3 expression is one of the first novel immunohistochemical markers predictive of overall worse survival rates in PDTC patients [16, 21].

## 4. Molecular Genetics

Genetic alterations in thyroid follicular cells, such as point mutations and translocations, increase the mitogenesis and pathogenesis of thyroid carcinomas. Therefore, appreciating the molecular biology of PDTCs could help develop better-targeted therapeutic modalities and more accurate diagnoses. If left untreated, PDTCs (whether they arise de novo, or from PTCs or FTCs) could eventually progress by dedifferentiation to ATCs (Figure 2). Interestingly, according to the literature, those three pathways leading to PDTCs are invariably orchestrated by varying genetic alterations [16]. 

**Figure 3:**
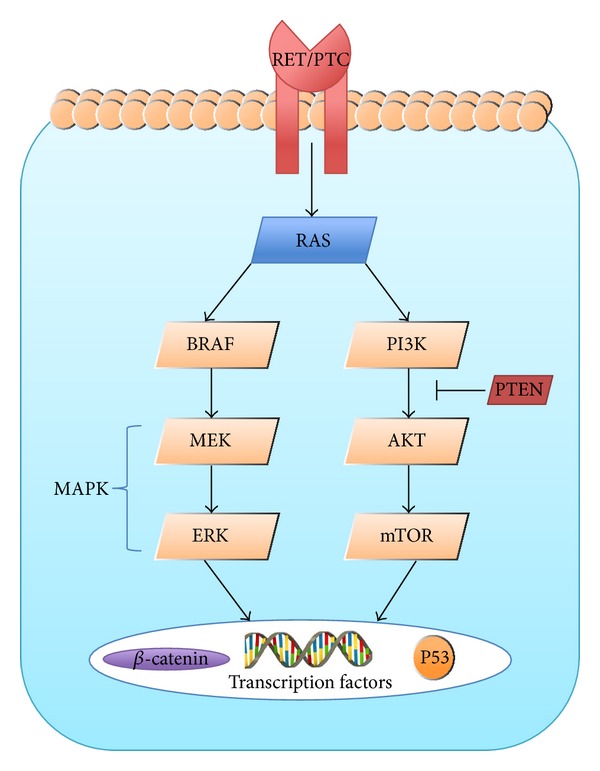
This diagram summarizes the signaling pathway in thyroid carcinomas.

Genetic alterations of follicular cells that lead to carcinogenesis are caused by unopposed activation of either the mitogen-activated protein (MAP) kinase pathway or the phosphatidylinositol-3-kinase (PI3K)/AKT pathway [1]. Specifically, the MAP kinase pathway (encompassed by the MEK and ERK kinase cascade) is regulated by the RET, RAS, and BRAF genes. Point mutations in the BRAF and RAS genes or RET/PTC translocation can lead to unopposed cellular proliferation and to a carcinogenic environment via the MAP kinase pathway [1] (Figure 3). 

*BRAF gene* alterations (commonly, valine-to-glutamate substitution at codon 600) are present in about 30% to 45% of patients with PTCs and, on average, in 15% (range, 12% to 47%) of patients with PDTCs [1, 16, 18]. Follicular carcinomas are not susceptible to BRAF mutation; therefore, PDTCs arising from FTCs must be BRAF-mutation-negative. The BRAF gene is a marker of adverse prognostic factors, including disease aggressiveness, tumor recurrence, lymph node or distant metastatic disease, and extrathyroidal extension [1, 18]. Interestingly, thyroid carcinomas with BRAF-mutations are also associated with a decreased capability to trap radioiodine (^131^I) [18]. 

*RAS gene* alterations (commonly, point mutations at HRAS codon 12 or 61 and NRAS at codon 13) are present in about 40% to 50% of patients with FTCs and, on average, in 35% (range, 20% to 40%) of patients with PDTCs [1, 16, 18, 22]. Unlike the BRAF gene, which is specific to the activation of the MAP kinase pathway, RAS can activate both the MAP kinase pathway and the PI3K/AKT pathway. Specifically in patients with PDTCs, oncogenic RAS activation is a prevalent genetic alteration—and a marker of tumor dedifferentiation and adverse prognostic outcome [10, 18]. Of note, RAS mutations stimulate chromosomal instability and, thus, may predispose to tumor dedifferentiation, perhaps explaining the increased prevalence of mutant RAS in patients with ATCs [16, 18]. However, Nikiforov et al. noted that mutant RAS is unlikely to be solely capable of driving tumor dedifferentiation, given its high prevalence (45%) in patients with pure WDTC and in those with benign thyroid adenomas [16]. Conversely, Garcia-Rostanet al. stated that histologic dedifferentiation is not necessarily driven by BRAF or RAS mutations individually, but rather represents the cooperation of multiple genetic alterations that likely stimulate dedifferentiation [10]. 

*TP53 gene* alterations (point mutation at codon 273 leading to the inactivation of p53) are rarely associated with WDTCs; however, they are highly prevalent in patients with PDTCs (about 28%; range, 17% to 38%) and in patients with ATCs (64%; range, 20% to 88%) [18, 23, 24]. Termed the “molecular policeman” [1], p53 broadly has three principal functions that preferentially hinder, and possibly reverse, carcinogenic effects: quiescence, senescence, and apoptosis [1]. Interestingly, in histological samples of a tumor containing both WDTC and PDTC components, alterations in the p53 gene were circumscribed to the less differentiated component [24]. These findings suggest that, unlike the RAS and BRAF gene alterations, p53 mutations possess an exclusive function in triggering tumor dedifferentiation and evolution to PDTC and ATC [23, 24].

*RET/PTC* rearrangements (paracentric inversion of chromosome 10 or a reciprocal translocation between chromosomes 10 and 17), ultimately result in the unopposed activation of the MAP kinase pathway. Most commonly, RET gene rearrangements are exclusively expressed in patients with PTCs, usually those with a history of radiation exposure (e.g., from the 1986 nuclear power plant accident at Chernobyl) [1, 18, 23]. Such rearrangements are almost never expressed in patients with PDTCs [16, 18]. 

*PAX8 : PPAR*γ** rearrangements, (chromosomal translocation of (2;3)(q13;p25)) resulting in the fusion of the PAX8 gene and the peroxisome proliferator-activated receptor gene, are almost always associated only with follicular carcinomas [1, 18]. Such rearrangements are almost never expressed in patients with PDTCs [16, 18]. 

Table 1 summarizes the prevalence of genetic alterations in patients with various thyroid carcinomas.

## 5. Clinical Presentation

Of all thyroid carcinomas worldwide, PDTCs account for only 4% to 7% [17]. They are typically diagnosed in patients between the age of 55 and 63 years, a 2 : 1 female predominance [16]. Interestingly, a regional variance has been noted for instance, northern Italy (an endemic goiter region) has incidence rates as high as 15%, whereas the rates are much lower in the United States (1.8%) and Japan (<1%) [2, 16]. These discrepant rates suggest that the causes of PDTCs are multifactorial, including genetic, environmental, and dietary causes [19]. 

Consistent with their intermediate differentiation, PDTCs display clinical behaviors of intermittent biological aggressiveness. When diagnosed, PDTCs are typically already at an advanced stage of disease, with extrathyroidal extension and extensive local invasion [8, 16]. They have a propensity to metastasize to regional lymph nodes (50% to 85 %), and distantly (36% to 85%), most commonly to the lung (14% to 54%) and bones (18% to 33%) [2, 25]. Furthermore, the 5-, 10-, and 15-year survival rates are considerably lower in patients with PDTCs (50%, 34%, and 0%) than in patients with WDTCs (95%, 86%, and 81%) [2, 8, 14]. Several retrospective studies found that the following factors negatively correlate with survival rates: patient age >45 years, tumor size >4 cm, lack of radioactive iodine therapy postoperatively, cervical lymph node involvement, tumor necrosis, mitotic index >3 per 10 high-power fields, local recurrence, and distant metastasis at time of diagnosis [4, 10, 19, 26].

The initial workup typically includes an ultrasound examination (to evaluate the thyroid gland and cervical lymph node compartments) and FNA sampling (for cytologic analysis). In patients with suspected PDTCs, preoperative evaluation of vocal cord function is critical, in light of the high rates (50% to 75%) of extrathyroidal invasion [2]. The recurrent laryngeal nerve is frequently affected in patients with extrathyroidal disease. Randolph and Kamani found that vocal cord paralysis was the most reliable clinical marker (sensitivity, 76%; specificity, 100%) of invasive thyroid carcinomas [27]. In their study, preoperative laryngoscopy revealed that 70% of patients with invasive disease presented with vocal cord paralysis, as compared with only 0.3% of patients with noninvasive disease [27]. They noted that preoperative vocal cord paralysis involvement should be a presumptive diagnosis of invasive disease.

If extrathyroidal invasion, substernal extension, or distant metastasis is suspected, axial imaging with computed tomography or magnetic resonance imaging is important in operative planning. If extrathyroidal extension is suspected, further studies (including esophagography, esophagoscopy, and bronchoscopy) may be required to assess neighboring structures.

## 6. Treatment Modalities

Given the infrequency of PDTCs and the previously lack of standard diagnostic criteria, no standard guidelines currently exist for the management of PDTCs. However, most endocrine surgeons agree that the primary treatment is a total thyroidectomy, with lymph node dissection whenever feasible [14, 16, 19]. Postoperative options such as radioactive iodine (RAI) (^131^I) ablation therapy, external beam radiation therapy (EBRT), and chemotherapy remain poorly established. 

*RAI (* ^131^ *I)* ablation therapy postoperatively is controversial. Sanders Jr. et al. recommended, in light of the potential for PDTC uptake and the lack of major side effects, considering ^131^I therapy in all postoperative patients who underwent a complete resection [2]. PDTCs have demonstrated the capability to uptake ^131^I in up to 80% to 85% of patients, yet studies have not statistically shown that ^131^I prolongs 5-year survival [19]. Moreover, since over 15% of patients with PDTCs have an BRAF mutation, which is associated with a decreased capacity to trap radioiodine (^131^I) [18], the value of RAI ablation in this population is limited.

*EBRT* is typically reserved for patients with aggressive forms of PDTCs or for patients whose resection was incomplete, with remnants of malignant disease retained in the neck postoperatively [2, 16, 19]. Extrapolating from poorly prognostic WDTC radiation studies, Sanders et al. recommended considering EBRT in patients with PDTCs who meet at least 1 of these criteria: (1) tumors >4 cm with minimal extrathyroidal extension without distant metastasis (i.e., extension to the sternothyroid muscle or perithyroid soft tissues); (2) extensive extrathyroidal extension of any tumor size (i.e., extending past the thyroid capsule into the subcutaneous soft tissues, larynx, trachea, esophagus, recurrent laryngeal nerve, or mediastinal vessels); and/or (3) regional lymph node metastasis [2, 28]. The utility of adjuvant EBRT is questionable: retrospective studies involving EBRT in patients with PDTCs showed no statistical improvement in overall survival rates [2, 19].

*Chemotherapy* has clinically been reserved for patients with inoperable PDTCs. It has some clinical practicality in patients with ATCs. An intensive chemotherapy regimen can aid in locoregional control by improving resectability or by reducing disease progression [2, 16]. 

*Chemoradiation* is currently undergoing experimental review for any possible benefit in patients with PDTCs [19]. However, to date, survival rates in patients with PDTC have not improved after chemotherapy, either alone or in combination with radiotherapy.

Perhaps the most promising PDTC treatments will come from advancements in molecular inhibitors targeting the TP53 gene, the MAP kinase pathway, and/or the PI3K/AKT pathway [16, 18]. Novel therapeutic agents, such as sorafenib, vandetanib, sunitinib, and motesanib, are currently undergoing promising clinical trials. These agents can selectively target multiple kinase pathways like vascular endothelial growth factor (VEGF), which is important in angiogenesis. They also target RET, BRAF, and/or protein derived from the RET/PTC realignment [29]. In the near future, these molecular inhibitors might be administered (alone or in conjunction with other treatment modalities) to potentiate treatment options for patients with PDTC [18, 29].

## 7. Follow-Up Imaging

Traditionally, RAI imaging has been used to assess patients with PDTCs [14, 30]. Most WDTCs retain the capacity to take up iodine, so the use of RAI imaging is suitable in patients with WDTCs. In contrast, PDTCs have a reduced capacity to trap RAI [18]; so the use of RAI imaging in patients with PDTCs can falsely suggest a complete resection [2, 30]. 

The “flip-flop” theory postulates that as less differentiated malignant cells lose their ability to take up RAI, they increase their ability to take up fluorodeoxyglucose (FDG) [30]. Recent statistically sound studies have noted the value of FDG-positron emission tomography (PET) scans in localizing occult disease and in increasing the rates of early detection of recurrent or metastatic PDTCs [14, 30]. In a multivariate analysis, Wang et al. determined that the strongest single predictor of survival and prognosis was the volume of disease as detected by FDG-PET. FDG-avid tumors with volumes over 125 mL positively correlated with poorer short-term survival [30]. 

Furthermore, Ito et al. recommended regular postoperative examinations in patients diagnosed with PDTCs, not only to predict prognosis but also to allow for timely postoperative care [31].

## 8. Conclusion

A limitation of our review (as well as a clinical challenge) is the lack of an accepted, unified set of criteria for characterizing PDTCs. The 2006 Turin proposal, albeit criticized as overly restrictive by some investigators, at minimum offers a comprehensive, homogenous set of diagnostic criteria, paving the way for increasing our understanding of the pathogenesis and treatment of this disease. Perhaps with increased specificity in immunohistochemistry and molecular genetics tests, our definition of PDTCs will become more standardized and it can more readily be diagnosed. These tumors, though infrequent, do have distinct clinicopathologic features; pathologists and clinicians alike must know how to recognize and diagnose them. Clinical management and recurrence detection are still in their infancy for this disease, but advances in molecular genetics continue to promise a large role in the refinement of diagnosis, prognosis, and therapeutic modalities.

## Figures and Tables

**Table 1:** Prevalence of genetic alterations in patients with various thyroid carcinomas.

Altered gene	PDTC	PTC	FTC	ATC
RET/PTC	0%	20%	0%	0%
TP53	20–30%	0%	0%	65–70%
BRAF	15%	45%	0%	20–25%
RAS	30–35%	10–15%	45%	50–55%
*β*-catenin	20–25%	0%	0%	65%
PAX8 : PPAR*γ*	0%	0%	35%	0%

PDTC: poorly differentiated thyroid carcinoma.

PTC: papillary thyroid carcinoma.

FTC: follicular thyroid carcinoma.

ATC: anaplastic thyroid carcinoma.
